# Bathing

**Published:** 1870-10

**Authors:** 


### BATHING.

1. Bathe quickly, wipe dry, and walk off rapidly, all within ten minutes.

2. It is dangerous to bathe when tired or at bed time ; hence, it is better to make a rule to bathe before breakfast, when the system has been rested by a night's sleep.

3. Before bathing, wash the face, hands and head in cold water.

4. Do not bathe within two hours of eating a full meal ; death has often resulted from inattention to this rule.

5. Cold water baths are hurtful under all circumstances to very young or very old people ; to invalids, to consumptives, to those subject to spitting blood. It is the safest rule that a woman should never take a cold bath other than to rub the whole surface quickly with a soft towel, dipped in water pressed out ; lay the towel smooth on the hand, and rub quickly the whole body, within ten minutes.

The general health of mankind would be most benefited by avoiding all cold water or sea bathing, and take but one bath a week, and that in a room not under seventy degrees, on Saturday night, using warm water, soap, and a common new scrubbing-brush, bristles at least three-quarters of an inch long; wet the body all over with water; then rub a piece of soap over the brush, and with it rub the body with a will, as far as can be reached in every direction, rapidly; then rinse off and wipe dry with a cotton towel at least a yard square; this leaves the skin more perfectly dry than common linen or crash towel; the whole operation should be performed within ten minutes; the water should be at least eighty degrees; this kind of bathing certainly cleanses the skin, stimulates the surface, and leaves the body in a safe condition.

Temperature for baths—cold bath, 50°; tepid bath, 70°; warm bath, 90°; hot bath, 110°; vapor bath, 130°.

